# Matrons in Council: V. The Elements Must Be in the Woman

**Published:** 1917-09-01

**Authors:** 


### THE MATRONS' AND SISTERS' DEPARTMENT.

### MATRONS IN COUNCIL.

### V. The Elements Must be in the Woman.

A great deal is being said and written at the present time about the education of nurses in order to secure a better all-round nurse at the finish. Education, in the limited sense of the word—book learning—will never make a nurse, the elements of making the true nurse must be *in* the woman. The bottom of the whole question is home training; women who have had good mothers, who have taught them obedience and self-discipline with a thorough domestic training, are the women who will make the best nurses; no amount of hospital training and discipline will make up for the lack of the proper foundation; given the good foundation, then, we can go on with the education, and we must be careful not to build carelessly on that foundation.

We want to do nothing which will take away from the English nurse the title of being the best of all practical nurses, though often inferior in theory to her Colonial or American sister.

The practical training in our medium-sized hospitals usually turns out a better nurse than the nurse of the larger hospital, and this may be accounted for by the fact that nearly all smaller hospitals have to get in their sisters from varied training schools, so often combining the best of many hospitals in one school. This variety of training can be developed in various ways; for example, no nurse should be made a sister in her hospital till she has held a post elsewhere; and hospitals might combine to give training. This will have to be if our under-fifty-bed hospitals have to be nursed and yet get nurses for training; this combining of hospital training is dreaded by all, as all the unknown is. The matron of the large hospital does not want to have to take probationers from small hospitals without a say in the choice of the same, just as the matron of the small hospital wants still to choose her own probationers; this difficulty could be overcome by a certain number of small hospitals being affiliated to the large hospitals and the matrons of all to form a committee for the choosing of probationers. A very high standard of ideals could then be promulgated, and it would no doubt be the aim of each matron to see that her nurses turned out the best women.

It would be well if boards of management could arrange that more servants were kept, so that nurses had not so much of the domestic work to do. A certain amount is most essential in order to teach the nurse the importance of cleanliness, but the cleaning of brasses and the scrubbing of lockers is not really essential, and could be done by a skilled worker in another branch.

It is necessary that the hours of duty be curtailed. This is a very difficult matter, as it means the expenditure of more money in building larger nurses' homes in order to make room for a larger staff. If one day a week off duty could be arranged, as well as the present daily off duty, we should find a great deal of benefit, both to nurses and also to patients.

The pay of nurses generally should be very considerably raised, and nurses should be helped as far as possible by their matrons in the wise expenditure of their work, time, and money; this does not mean that the matron is to interfere, but by friendly intercourse can often guide and direct her staff into higher ideals by which to use all their talents. The autocratic matron is very much out of date; most women in the position of matron wish to be on friendly terms with their nurses.

Whatever the curriculum or the examination set up by the College may be, a certain definite number of marks should be allotted to the matron of the nurses' training school, so that nurses will continue to realise that their practical work during their training is to count a good deal before they become certified nurses. If the hospitals would all amalgamate with the College so that the College examination is taken as the final examination of the hospitals, then we could get a thoroughly well-trained theoretical nurse, who at the same time is guaranteed a practical nurse by the combination of the College and hospital marks.

Whatever is done in the way of theoretical teaching, let us always endeavour to place more clearly before our nurses the highest possible reasons why they should undertake and continue in the nursing of the sick.

